# Summer rainfall over the southwestern Tibetan Plateau controlled by deep convection over the Indian subcontinent

**DOI:** 10.1038/ncomms10925

**Published:** 2016-03-07

**Authors:** Wenhao Dong, Yanluan Lin, Jonathon S. Wright, Yi Ming, Yuanyu Xie, Bin Wang, Yong Luo, Wenyu Huang, Jianbin Huang, Lei Wang, Lide Tian, Yiran Peng, Fanghua Xu

**Affiliations:** 1 Ministry of Education Key Laboratory for Earth System Modeling, Center for Earth System Science, and Joint Center for Global Change Studies (JCGCS), Tsinghua University, Room S813, MengMinwei Science Building, Qinghuayuan 1, Beijing 100084, China; 2 Geophysical Fluid Dynamics Laboratory, Princeton/NOAA, Princeton, New Jersey 08540-6649, USA; 3 State Key Laboratory of Numerical Modeling for Atmospheric Science and Geophysical Fluid Dynamics, Institute of Atmospheric Physics, Chinese Academy of Sciences, Beijing 100029, China; 4 Key Laboratory of Tibetan Environment Changes and Land Surface Processes, Institute of Tibetan Plateau Research, Chinese Academy of Sciences (CAS), and the CAS Center for Excellence in Tibetan Plateau Earth Sciences, Beijing 100101, China

## Abstract

Despite the importance of precipitation and moisture transport over the Tibetan Plateau for glacier mass balance, river runoff and local ecology, changes in these quantities remain highly uncertain and poorly understood. Here we use observational data and model simulations to explore the close relationship between summer rainfall variability over the southwestern Tibetan Plateau (SWTP) and that over central-eastern India (CEI), which exists despite the separation of these two regions by the Himalayas. We show that this relationship is maintained primarily by ‘up-and-over' moisture transport, in which hydrometeors and moisture are lifted by convective storms over CEI and the Himalayan foothills and then swept over the SWTP by the mid-tropospheric circulation, rather than by upslope flow over the Himalayas. Sensitivity simulations confirm the importance of up-and-over transport at event scales, and an objective storm classification indicates that this pathway accounts for approximately half of total summer rainfall over the SWTP.

## Abstract (web summary)

While precipitation over the Tibetan Plateau is a vital resource for glacier mass balance, river runoff and local ecology, the controlling mechanisms are poorly understood. Here, the authors combine observations and simulations and show that convective storms over India sweep moisture up and over the plateau.

## Graphical abstract

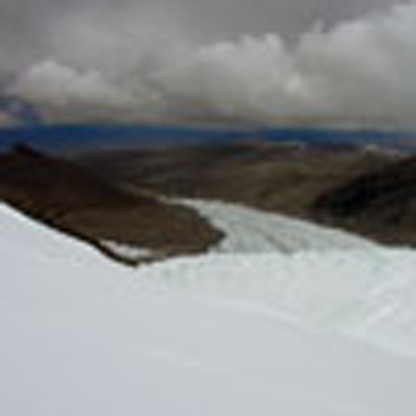

The Tibetan Plateau (TP), sometimes referred to as ‘the world's water tower'^1^, is a critical region for water resources in Asia. Nearly one sixth of the global population depends on rivers with headwaters on the TP, and changes in precipitation over the TP have great impacts on glaciers, river discharge, plant phenology and natural hazards^1^,^2^,^3^,^4^,^5^. Summer (June–September) precipitation, which occurs mainly in convective storms (CSs), accounts for 60–90% of total annual precipitation over the TP and surrounding areas^6^,^7^,^8^. Trends and variability in summertime precipitation over the TP are therefore likely to exert strong influences on climate and socio-economic conditions throughout many parts of Asia.

The spatiotemporal variation of summer precipitation over the TP is complex, and is mainly regulated by a combination of summer monsoons and regional moisture recycling^2^,^3^,^9^,^10^. Rainfall over the eastern TP has been extensively studied and well-documented, with a relative abundance of ground measurements, and is closely linked to the South Asian monsoon via a moisture corridor that extends along the Brahmaputra River from the Bay of Bengal^9^,^11^,^12^. However, despite recent sharp declines in glacier extent^2^,^3^, the southwestern TP (SWTP) has received considerably less attention. The SWTP is a semiarid region with a vulnerable ecosystem, and precipitation systems over the SWTP often serve as precursors of synoptic systems downstream^4^,^6^,^11^. Some convective instabilities that develop over the SWTP cause extreme rainfall and severe flooding in East Asia as they develop and move eastward^9^,^13^.

The factors that control precipitation variations over the SWTP remain poorly understood. Here we establish that summer rainfall over the SWTP and CEI is closely related via a previously unreported ‘up-and-over' moisture transport path, in which moist, cloud-laden air lifted by convective systems over CEI is swept over the SWTP by the mid-tropospheric circulation. This relationship is evident in both observational data sets and global climate model simulations, and depends on the coincident occurrence of convective systems over CEI and southwesterlies in the middle and upper troposphere over the Himalayas. Several lines of evidence confirm both the existence of the up-and-over moisture transport path and its importance in the water budget of the SWTP, including satellite measurements, isotopic ratios in precipitation, numerical sensitivity simulations and backward trajectory calculations. The results consistently indicate that up-and-over transport accounts for at least half of total summer rainfall over the SWTP, and show that this pathway dominates moisture supply in this region. The SWTP will be substantially drier without the up-and-over transport associated with convective systems over CEI.

## Results

### Linkage of summer rainfall between SWTP and CEI

**Figure 1:**
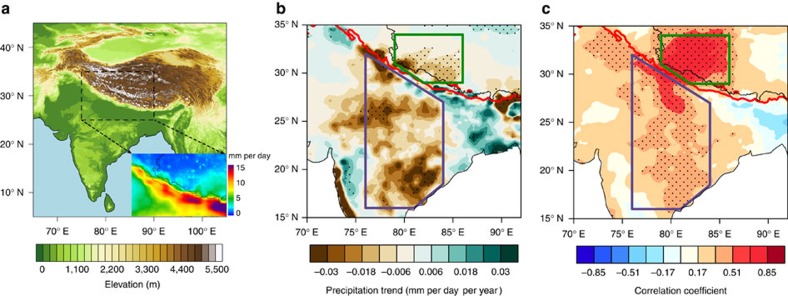
June–September precipitation characteristics. (**a**) Topography of the Tibetan Plateau and surrounding areas. The inset shows summer precipitation averaged over 1998–2013 using TRMM. (**b**) Precipitation linear trends during 1951–2007 using APHRODITE. (**c**) Correlation coefficient between area-averaged precipitation over the southwestern Tibetan Plateau (SWTP; green polygon) and precipitation over the entire study region (1951–2007, APHRODITE). Dots in **b**,**c** indicate statistically significant areas (*P*<0.01, using a two-sided *t*-test). Purple polygon in **b**,**c** indicates central-eastern India (CEI). The red isoline in **b**,**c** is the 2,500 m elevation contour locating the Tibetan Plateau.

**Figure 2:**
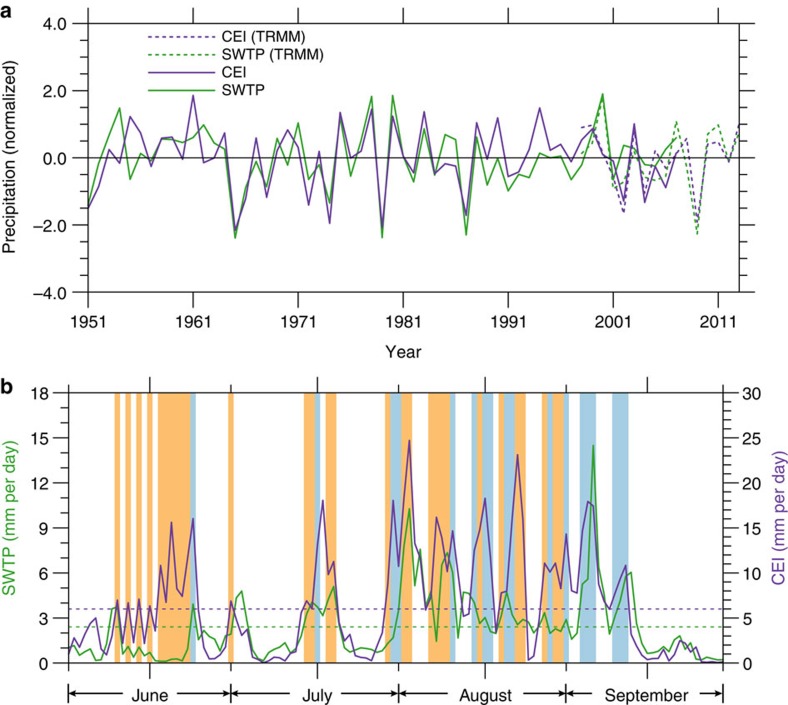
Summer precipitation variations. (**a**) Time series of standardized anomalies in summer mean precipitation over the southwestern Tibetan Plateau (SWTP; green) and central-eastern India (CEI; purple) using APHRODITE (1951–2007) and TRMM (1998–2013). (**b**) As in **a** but for daily precipitation from TRMM during June–September 2002. The light blue shading marks days with intrusive CSs, while the light orange shading marks days with non-intrusive CSs. Green dashed lines indicate the seasonal mean precipitation over the SWTP and purple for 6 mm per day over CEI.

Evidence of a strong connection between rainfall over the SWTP and rainfall over the Indian subcontinent emerges from historical records of precipitation (Fig. 1; see Methods for details). In particular, despite a large difference in rainfall magnitude across the Himalayas (Fig. 1a), significant decreasing trends have been observed over both the SWTP and central-eastern India (CEI; Fig. 1b). Decreases in rainfall over the Indian subcontinent over the past six decades have been attributed both to natural variability^7^,^14^ and to anthropogenic forcing^15^,^16^,^17^. Summer rainfall variability over the SWTP is highly correlated with summer rainfall variability over CEI (Fig. 1c). Indeed, summer precipitation over CEI is a reliable predictor for summer precipitation over the SWTP (Fig. 2a), with correlations of *r*=0.68 for APHRODITE^18^ data (based on rain gauge measurements during 1951–2007; *P<*0.001) and *r*=0.64 for TRMM^19^ data (during 1998–2013; *P<*0.01). The correlation between TRMM and APHRODITE estimates of precipitation exceeds 0.85 during their period of overlap (1998–2007; *P<*0.01) with a difference of <8% between their mean magnitudes. Strong correlations between precipitation variability over these two regions are likewise evident in two other observational data sets, as well as in simulations of historical climate and scenario-based projections of future climate by many global climate models (Supplementary Fig. 1). These results indicate a close and robust relationship between summer rainfall over the SWTP and summer rainfall over CEI that transcends data sources and model configurations. Quantitative differences in the correlation coefficients may be tied to differences in model physics, dynamics or resolutions, and are not explored here.

The strong relationship between summer rainfall over the SWTP and summer rainfall over CEI is somewhat surprising, as these two regions are separated by the Himalayan mountain range. The Himalayas are on average more than 5,000 m above mean sea level between 75 and 95° E, and act as a natural barrier for northward water vapour transport into the SWTP. Northward flow impinging on the Himalayas is mechanically lifted by the orography, with the southern foothills of the Himalayas among the most prominent regions of orographic precipitation on Earth^20^,^21^. This situation creates a strong precipitation gradient in the lee of the Himalayas. The sharp decrease in precipitation on the leeside of the mountain range suggests that the Himalayas strictly limit upslope moisture transport under stably stratified conditions. In other words, these mountains are sufficiently high that condensation during upslope flow ‘wrings out' most of the water, leaving little moisture available for precipitation in the interior of the SWTP. However, the interior of the SWTP experiences more precipitation than other similar places blocked by high mountain ranges, such as desert areas in the southwestern United States and South America. Furthermore, numerical modelling experiments^9^ and analyses of the isotopic composition of rainfall^22^,^23^,^24^,^25^,^26^,^27^ suggest that much of the rainfall over the SWTP during boreal summer can be traced back to the nearby ocean. The strong connection between the two regions must therefore be associated with alternative routes for moisture transport, rather than direct upslope flow. These results call for a re-evaluation of moisture transport into the SWTP and a careful analysis of how the relationship between the SWTP and CEI is established.

### An up-and-over route for moisture transport into SWTP

Summertime rainfall over the Indian subcontinent is dominated by deep CSs^10^,^21^,^28^,^29^,^30^. These CSs originate over the surrounding oceans, and are then observed to move northward or northwestward in company with strong onshore moisture transport^31^. Individual CSs may organize and develop into monsoon depressions, which in turn contribute significantly to monsoon rainfall over the northern part of the Indian subcontinent^32^,^33^. CS development preferentially occurs when monsoon southwesterlies and high-latitude northwesterlies converge and ascend along the southwestern flank of the Himalayas^13^.

Almost every large precipitation event over the SWTP occurs in tandem with a corresponding event over CEI. For example, precipitation over these two regions during summer 2002 (Fig. 2b) exhibits similar variations at both daily and weekly time scales (*r*=0.72, *P<*0.01). Variations at these time scales correspond to episodes of strong rainfall; for example, between June and September 2002, the same 34 days accounted for >60% of the total rainfall over both regions. The co-variability of precipitation over the two regions is thus primarily determined by the correspondence of individual convective precipitation events. We propose that this link manifests as strong transport of moisture and hydrometeors at middle-to-upper levels of the troposphere: moist, cloud-laden air is elevated by deep CSs over the Indian subcontinent and then advected over the Himalayas into the SWTP by the mid-tropospheric circulation. We term this transport pathway the ‘up-and-over' route.

### Intrusive and non-intrusive deep CSs

Not all precipitation events over CEI are accompanied by strong precipitation over the SWTP. In some cases, convective precipitation over CEI is confined to the south side of the Himalayas. We therefore categorize CSs over CEI into two types based on the characteristics of the mid-tropospheric flow near the Himalayas (see Methods). We define each CS as intrusive if the mid-tropospheric circulation permits it to influence the SWTP, and non-intrusive if its effects are confined to the south side of the Himalayas. Supplementary Figure 2 shows examples of typical intrusive and non-intrusive CS events. Both examples were characterized by a closed low-level (850 hPa) centre of low pressure south of the Himalayas; however, strong rainfall extended northward into the SWTP for the intrusive CS and did not for the non-intrusive CS. The northward extension of rainfall in the intrusive case was associated with strong cross-barrier (southwesterly) winds of up to 10 m s^–1^ at 500 hPa and a region of persistent large-scale ascent that extended from the Indian subcontinent to the SWTP. At 300 hPa, strong southwesterlies ahead of an approaching upper level trough pushed the upper tropospheric high southeastward. These middle and upper tropospheric circulation patterns promoted the advection of convectively lifted moisture and hydrometeors from the Indian subcontinent northeastward over the SWTP, in stark contrast to the circulation patterns observed during the non-intrusive CS. In the non-intrusive case, the low-pressure system extended upward from the surface to 500 hPa, with predominant easterly flow along the mountain range. Meanwhile, the centre of high pressure at 300 hPa straddled the mountain range, with northwesterly flow towards the SWTP and northeasterly flow towards the Indian Subcontinent. The absence of cross-barrier flow in the middle and upper troposphere (500 and 300 hPa) effectively prevented the northward transport of moisture and hydrometeors, so that rainfall was mostly confined to the Indian subcontinent. The moist static energy at 300 hPa was likewise much larger during the intrusive case than during the non-intrusive case, indicating that upper levels of the troposphere experienced greater moistening and diabatic heating during the intrusive case.

### WRF simulations and backward trajectory calculations

It is difficult to distinguish the relative contributions of up-and-over transport, upslope transport and local processes using observations alone. For example, the pattern of precipitation we attribute to up-and-over transport might instead be generated by two separate rainfall events over the SWTP and CEI. We therefore conduct a series of sensitivity simulations using the Weather Research and Forecasting (WRF) model (see Methods), in which we estimate the quantitative contributions of up-and-over transport, upslope transport and local recycling processes to the precipitation over the SWTP by reducing the advection of moisture and hydrometeors across the southern boundary into the SWTP within certain layers (Supplementary Fig. 3). A sensitivity simulation with hydrometeor advection entirely disallowed gives negligible precipitation change over the SWTP as compared with the control simulation (Figure not shown); however, this does not necessarily indicate that hydrometeor transport is unimportant given the large uncertainties in model simulated hydrometeor fields. In light of these uncertainties, we focus on the moisture transport within different layers for the following sensitivity simulations. We assume that water vapour advection across the southern boundary of the Plateau over CEI initially located more than 5 km above ground level (AGL) is mainly related to up-and-over transport, while advection of CEI water vapour initially located below 2.5 km AGL is mostly associated with upslope transport. Advection of CEI water vapour between 2.5 and 5 km AGL is assumed to be a mixture of the up-and-over and upslope transport. We use transport in this layer to represent uncertainties associated with partitioning between the two pathways.

**Figure 3:**
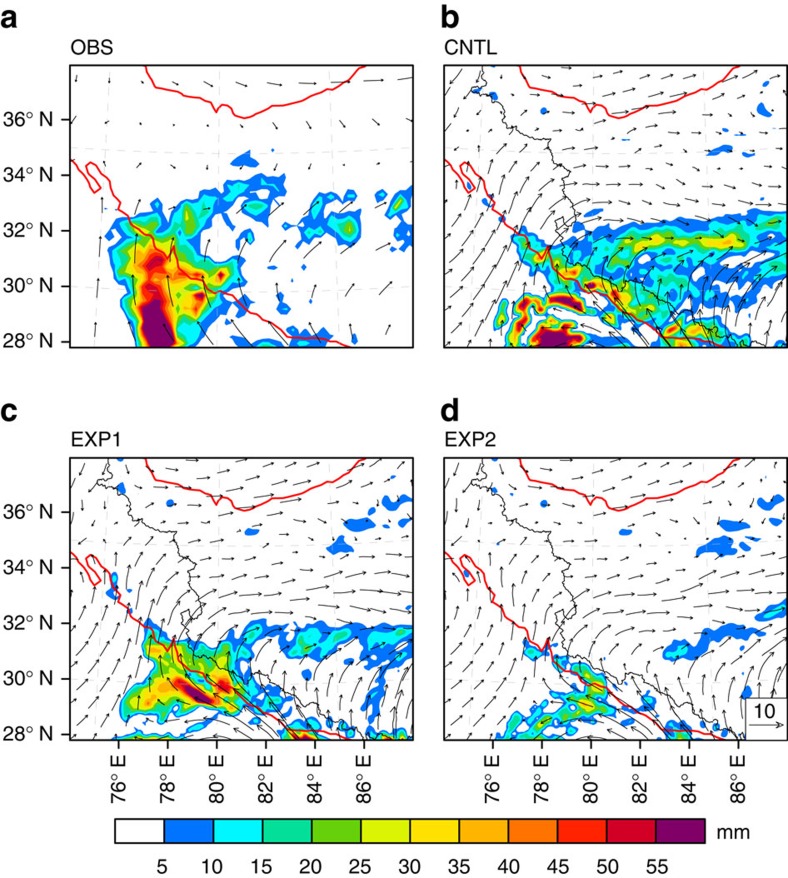
Sensitivity simulations using WRF. The distribution of precipitation overlaid with 500 hPa winds from (**a**) observations and reanalyses (precipitation from TRMM and wind fields from ERA-Interim), (**b**) the control simulation of the intrusive rain event on 12 September 2002, (**c**) a simulation of the same case but with advection of moisture above 5,000 m AGL reduced by 50% and (**d**) a simulation of the same case but with advection of moisture above 2,500 m AGL altitude reduced by 50%. The red isoline is the 2,500 m elevation contour.

The distribution of precipitation and the upper level circulation produced by the control simulation agree well with TRMM observations and ERA-Interim reanalyses (Fig. 3). Simulated precipitation over the SWTP decreases by ∼56% when advection of upper layer (>5 km AGL) water vapour is reduced by half. Halving the transport of water vapour at levels >2.5 km, AGL further reduces the precipitation, with simulated rainfall only ∼18% of that produced in the control simulation. Simulated precipitation over the SWTP essentially disappears (∼2% of the control simulation) when water vapour transport across the southern boundary is halved through the entire vertical column. These results indicate that the vast majority of the precipitation in this case (∼98%) depends on transport of moisture from the Indian subcontinent, with large contributions (56–82%) attributable to up-and-over transport. Additional simulations for other intrusive CSs show similar results (Supplementary Fig. 4), indicating that these contributions are robust.

The topographic features of the Himalayas, including both valleys and ridges, are well-represented by the 9-km grid used for the WRF simulations (Supplementary Fig. 5). Moisture transport can be found both in the valleys and at middle-to-upper levels, indicating that the upslope and up-and-over routes coexist. However, the reduction of moisture transport in the sensitivity simulations is most pronounced when advection of upper layer moisture is eliminated, indicating that moisture supply during these events depended most critically on up-and-over transport.

Our conclusions regarding the importance of up-and-over transport during the 12 September 2002 intrusive case are further supported by backward trajectory calculations using the FLEXPART particle dispersion model^34^ (Supplementary Fig. 6). Trajectory-derived transport pathways preceding the rain event are qualitatively consistent with the results of the WRF simulations described above. The quantitative partitioning of ‘up-and-over' and ‘local convection' trajectories is very sensitive to the criteria used to define these subsets; however, reasonable criteria assign 50–75% of these particles to the up-and-over route, indicating that transport via the up-and-over route was at the very least comparable to local convection (including that dependent on upslope moisture transport) during this event. Overall, the evidence consistently supports both the existence and the critical importance of the up-and-over transport to SWTP precipitation at event scales.

### Moisture transport and isotopic observations

Both satellite measurements and reanalyses confirm the existence of mid-tropospheric moisture corridors extending from CEI into the SWTP associated with intrusive CSs (for example, Supplementary Figs 7 and 8). By contrast, the same moisture fields show distinct breaks near the Himalayas for non-intrusive cases (not shown). The consistency between AIRS retrievals and ERA-Interim estimates of water vapour in this case indicates that the reanalysis is able to adequately capture the circulation and moisture fields over this area, as AIRS radiances were not assimilated by ERA-Interim until April 2003 (ref. 35). The vertical structure of the reanalysis moisture fields and circulation along the 80° E longitude transect (Supplementary Fig. 8) during typical intrusive and non-intrusive cases shows significant differences in the vicinity of the Himalayas and the SWTP. During the intrusive case, moist air and condensate lofted by strong upward motion were advected northward to the SWTP in the 400–600 hPa layer. By contrast, advection at these levels was directed southward during the non-intrusive case. Upslope transport also tends to be enhanced during intrusive events (Supplementary Fig. 8), but the sensitivity simulations described above indicate that rainfall during this event depended primarily on simultaneous increases in up-and-over transport. Contrasts in upper level circulation and rainfall between these two cases are also reproduced by the high-Asia refined analysis (Supplementary Fig. 9).

**Figure 4:**
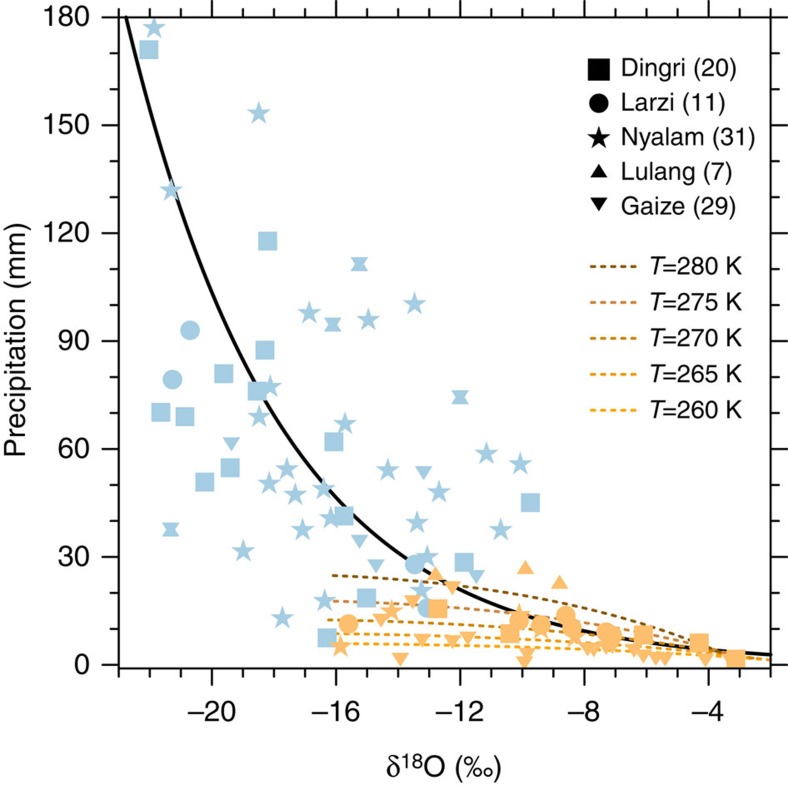
Isotopic content of precipitation. Scatter plot of summertime precipitation amount and δ^18^O in precipitation for 98 rainfall events at four TNIP observation stations and one ITP observation station during 1998–2007. Blue markers indicate values observed during intrusive CSs (59 events), while orange markers indicate value observed during non-intrusive CSs (39 events). The black solid line indicates the exponential trend line for all the markers. The coloured dashed lines indicate theoretical estimates of the amount effect in local convection with different initial temperature (ranges from 260 to 280 K) calculated by isotope theoretical model.

Additional evidence for the moisture source associated with intrusive CSs over the SWTP can be gleaned from the stable oxygen isotope ratio (δ^18^O) in precipitation. This tracer has been widely used to infer key characteristics of precipitation, including moisture source regions, transport history and the degree of rainout^24^,^25^,^26^,^27^. We use event-based observations of δ^18^O in precipitation from four stations in the Tibetan Network for Isotopes in Precipitation (TNIP) and one station operated by the Institute of TP Research (ITP) during 1998–2007 to evaluate the contributions of different moisture sources and transport pathways to summertime rainfall over the SWTP. A total of 98 summertime rainfall events (59 intrusive cases and 39 non-intrusive cases) were selected corresponding to the identified CSs (see Supplementary Table 3 for details). As shown by previous studies, precipitation amount and δ^18^O are inversely related in this region (Fig. 4), with a lower ratio of heavy isotopes during events with higher precipitation amounts^27^. Moreover, we find that intrusive CSs were consistently associated with larger precipitation amounts and lower values of δ^18^O than non-intrusive CSs. This difference is consistent with differences in moisture source and transport effects, but is also potentially consistent with the well-known amount effect, which also manifests as an inverse relationship between δ^18^O and precipitation amount^36^,^37^. To distinguish between these two possibilities, we generate theoretical estimates of the amount effect in local convection (dashed curves in Fig. 4; see Methods for details) using a model intended to represent rain events due to either upslope transport or local recycling (which both deliver moisture to the atmospheric surface layer above the SWTP). The theoretical estimates are consistent with the observations collected during non-intrusive cases (when local convection is expected to dominate), but diverge sharply from the observations collected during intrusive cases. Ratios of δ^18^O in summer precipitation over the SWTP for intrusive cases are much larger than expected based on the theoretical curves. Previous studies have likewise shown that water vapour detrained from tropical convective clouds is more enriched in heavy isotopes than expected based on theoretical estimates^38^. The isotopic observations collected during intrusive cases are therefore consistent with efficient moisture transport from the tropical ocean via the up-and-over pathway, and inconsistent with heavy convective precipitation that depends on moisture sources that deliver water vapour primarily to the surface layer (that is, upslope flow and local evapotranspiration).

### Composite and mean precipitation profiles

**Figure 5:**
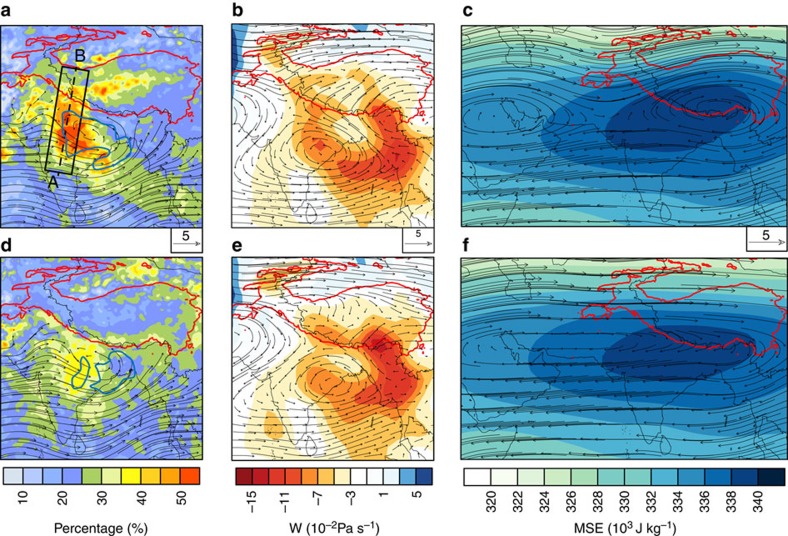
Composite precipitation and circulation characteristics. (**a**) Rainfall percentage overlaid with 850 hPa winds, (**b**) vertical velocity and winds at 500 hPa and (**c**) moist static energy and winds at 300 hPa for all intrusive CSs (227 events) during 1998–2005. (**d**–**f**) As in **a**–**c** but for non-intrusive CSs (229 events). The region is extended westward in **c**,**f** to show the Iran High. Blue contours in **a**,**d** indicate regions with >10 storm centres. The red isoline is the 2,500 m elevation contour. The black rectangle in **a** is used in subsequent transect calculations.

To extend our analysis beyond event scales, we construct composites for all intrusive and non-intrusive CSs during the period 1998–2005 (Fig. 5). Intrusive CSs account for approximately half of total summertime precipitation over both CEI (46%) and the SWTP (47%), with small interannual fluctuations. Precipitation occurs over the SWTP during 90% of the intrusive CSs, while rainfall is mostly confined to the Indian subcontinent during non-intrusive CSs. Non-intrusive CSs account for ∼35% of total summer rainfall over CEI, but with relatively little rainfall over the SWTP (17% of total summertime precipitation). Similar to the example cases discussed above, both composites have a low-level cyclonic circulation over CEI. CSs are more intense and extend further northwestward in the intrusive composite (Fig. 5a), while CSs and the monsoon trough are typically shifted slightly eastward towards the northwestern Bay of Bengal in the non-intrusive composite (Fig. 5d). The area of heavy rainfall during intrusive CSs extends towards the northeast in a band that stretches from CEI to the SWTP, but the area of heavy rainfall during non-intrusive CSs is smaller and mostly confined to CEI. Northward cross-barrier flow from CEI to the SWTP at 500 hPa is apparent in the intrusive composite (Fig. 5b), and contrasts with southward flow from the SWTP to CEI in the non-intrusive composite (Fig. 5e). An eastward centre of high pressure at 300 hPa straddles the southern periphery of the Plateau in the intrusive composite (Fig. 5c), while anticyclonic winds associated with the Iran High are much stronger and extend further eastward in the non-intrusive composite (Fig. 5f), suggesting that intrusive and non-intrusive CSs preferentially occur during the TP and Iranian Plateau modes of the upper tropospheric anticyclone, respectively^39^. This difference means that the upper level circulation over the SWTP during non-intrusive CSs is dominated by flow from the mid-latitudes rather than by flow from the Indian subcontinent. Moist static energy, which reflects the effects of convective heating and moistening, is likewise larger in the intrusive composite, with a southwest–northeast tilt that extends further over the SWTP^30^,^40^.

**Figure 6:**
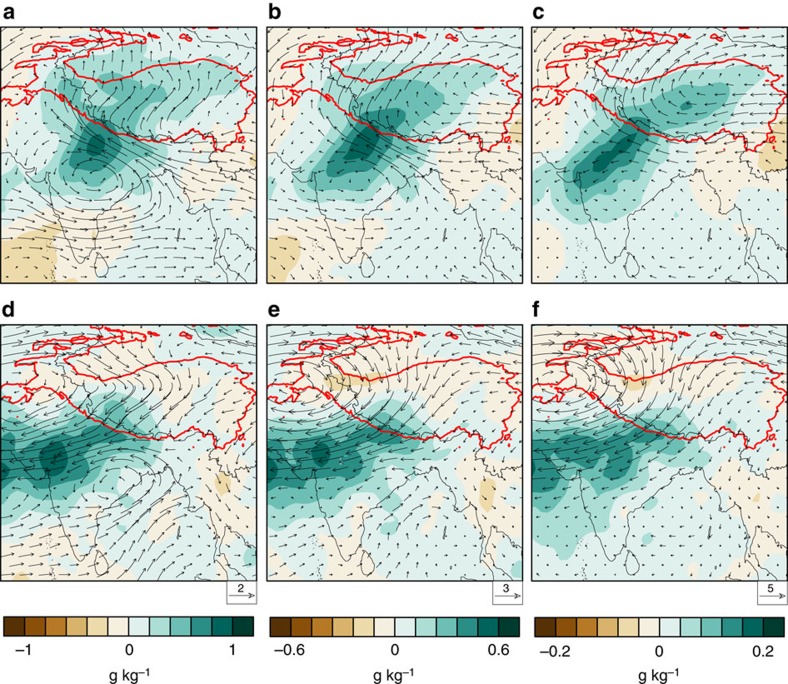
Composite moisture and circulation characteristics. Mid- and upper level moisture anomalies from AIRS overlaid with wind anomalies from ERA-Interim at (**a**) 500 hPa, (**b**) 400 hPa and (**c**) 300 hPa for all intrusive CSs (103 events) during 2002–2005. (**d**–**f**) As in **a**–**c** but for non-intrusive CSs (90 events). The red isoline is the 2,500 m elevation contour.

We apply the same method to create composite distributions of moisture anomalies in the middle and upper troposphere using twice-daily AIRS retrievals during 2002–2005 (Fig. 6). The intrusive composite features a tongue of positive moisture anomalies that extends northeastward from CEI to the SWTP in the middle and upper troposphere (Fig. 6a–c), consistent with the prevailing cross-barrier (southwesterly) flow. By contrast, positive moisture anomalies are confined mainly to the southern side of the Himalayas during non-intrusive CSs (Fig. 6d–f), consistent with the anomalous eastward extension of the Iran High and the associated anticyclonic flow. In summary, strong southwesterly flow promotes moisture transport over the SWTP during intrusive CSs, while strong northeasterly flow inhibits moisture transport over the SWTP during non-intrusive CSs.

**Figure 7:**
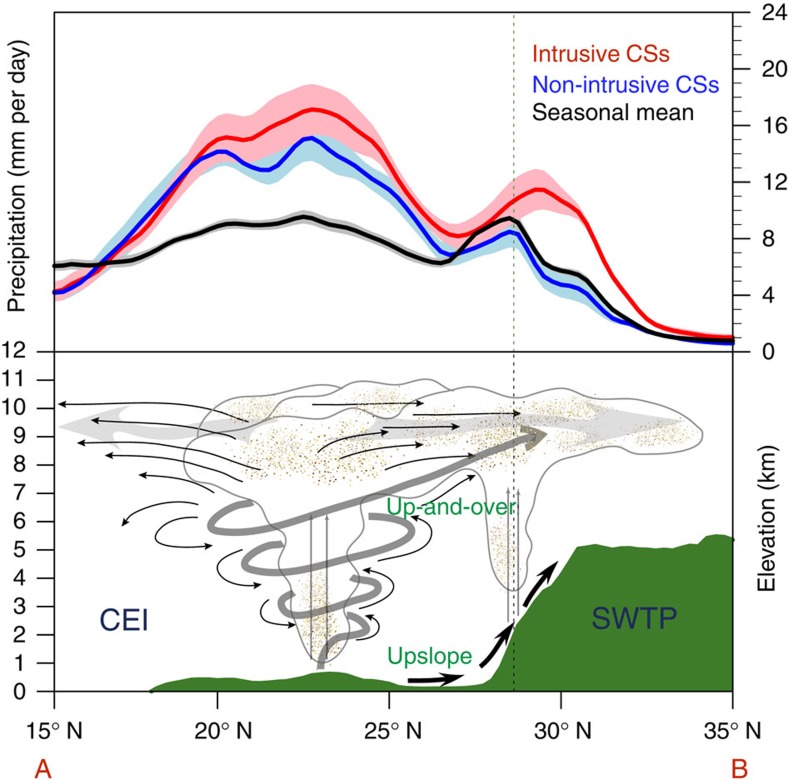
Precipitation profiles and a schematic of the up-and-over transport. Mean precipitation averaged over the black box marked in Fig. 5a for the intrusive CSs (red), non-intrusive CSs (blue) and total summer rainfall over 1998–2005 based on TRMM data. The shaded regions represent 1 s.e.m. The dashed line indicates the periphery of the southwestern Tibetan Plateau (SWTP), defined as topography 2,500 m above mean sea level.

Figure 7 shows mean precipitation along a transect perpendicular to the Plateau boundary for the intrusive and non-intrusive CS composites. Intrusive and non-intrusive CSs both result in large amounts of precipitation over the Indian subcontinent, but intrusive CSs produce much more precipitation over the SWTP than non-intrusive CSs. In particular, while orographic precipitation along the south slope of the plateau is apparent during both intrusive and non-intrusive CSs, precipitation extends northward into the Plateau during intrusive CSs but drops sharply at altitudes >2.5 km during non-intrusive CSs. This difference highlights the importance of up-and-over transport to precipitation over the SWTP. The up-and-over route is open during intrusive CSs, and enhances rainfall by delivering moisture and condensate to the inner part of the SWTP. By contrast, the up-and-over route is effectively closed during non-intrusive CSs, and precipitation rarely penetrates beyond the flank of the plateau.

## Discussion

Our results emphasize that mid-tropospheric advection of convectively lofted hydrometeors and moisture from CSs in CEI northward over the Himalayas (that is, ‘up-and-over' transport) is critically important for summertime precipitation variability over the SWTP. Relative to conventional upslope moisture transport, which is frequent but inefficient, up-and-over transport is more efficient but less frequent. Up-and-over transport occurs on ∼23% of days during summer and accounts for approximately half of total summer rainfall over the SWTP. Local convection also occurs frequently over the SWTP in summer, and follows a clear diurnal cycle with an afternoon peak. Episodes of local convection often follow intrusive CSs (Fig. 2b), when up-and-over transport is particularly strong. This lag may be explained by the importance of precipitation recycling processes^13^,^20^ in these episodes. Up-and-over transport, complemented by upslope transport, and associated rainfall represent the ultimate source of much of the moisture that participates in these recycling processes, and therefore contribute to total precipitation over the SWTP even on days when this transport is weak or non-existent. In this sense, up-and-over transport associated with intrusive CSs over India fundamentally determines the overall wetness of the SWTP: without this transport, the SWTP would be substantially drier. Although most precipitation over the SWTP occurs during summer, future studies should investigate the role of precipitation variability in other seasons (especially that associated with mid-latitude systems during winter) in the regional water budget.

We present strong evidence that intrusive CSs over the Indian subcontinent directly and significantly influence rainfall over the SWTP at both seasonal and event scales. This result has important implications for understanding and evaluating projections of future changes in regional precipitation to climate forcings, and for assessing the resilience of the biosphere, hydrosphere and cryosphere in this region. Confidence in projected changes in regional rainfall is notoriously difficult to achieve, but is vitally important for planning adaptation strategies, managing water resources and establishing environmental protections. Summer precipitation is projected to increase over both CEI and the SWTP under a warmer climate^41^, which would benefit ‘summer accumulation' type glacier growth in this region^3^. Although changes in both regions remain uncertain, the importance and robust nature of the relationship between these two regions establishes a framework for evaluating model performance in this region, eliminating unrealistic simulations and ultimately bolstering confidence in projections of future change.

## Methods

### Core data

APHRODITE^18^ is a long-term continental-scale daily gridded precipitation data set over Asia. These data have previously been used in studies of precipitation characteristics over both India^7^ and the TP. In addition to APHRODITE, we use observational estimates of precipitation from the TRMM Multi-satellite Precipitation Analysis^19^, GPCP^42^, CMAP^43^ and model output from CMIP5 historical simulations and future projections. Moisture fields are based on satellite retrievals from AIRS^44^,^45^ and reanalysis output from ERA-Interim^35^. ERA-Interim reanalysis and forecast fields are also used for dynamic and thermodynamic conditions in both the case study and the construction of composites. Additional details are provided in Supplementary Tables 1 and 2.

### Time series correlation coefficient

We calculate correlation coefficients using normalized anomalies (anomalies from the mean over the entire record divided by the s.d. over the entire record) of summer rainfall over CEI and the SWTP.

### CS identification and categorization

We identify and classify CSs using 3-h CLAUS^46^ and TRMM^19^ data. First, CSs over CEI are detected based on a conventional definition^13^ as contiguous areas larger than 10,000 km^2^ with CLAUS brightness temperatures (*T*_b_) colder than 219 K. Second, large rainfall events^8^ are identified as days with rainfall amounts larger than 6 mm per day. CS days are defined as days that satisfy both of the above criteria over CEI. We then further classify CS days into two types based on mid-tropospheric (600–400 hPa) wind directions within a region between 78 and 82° E longitude and 26 and 32° N latitude. CS days with northward flow stronger than seasonal mean (1 m s^–1^) in the mid-troposphere over the mountains are classified as intrusive, while CS days with southward flow in the mid-troposphere over the mountains are classified as non-intrusive. Finally, we use 850 hPa relative vorticity to track the location and intensity of each CS.

### WRF simulations

WRF model simulations are conducted using one-way nesting approach via the ndown approach (Detailed model setup is listed in Supplementary Table 4). A high-resolution (9 km) inner domain is nested within a coarse-resolution (27 km) domain (Supplementary Fig. 3), with the lateral boundary conditions of the inner domain obtained from hourly outputs from the outer domain simulations. This approach enables us to alter hydrometeor and water vapour fluxes at the boundaries of the inner domain. In addition to the control simulation, three other sensitivity simulations are conducted to differentiate and quantify the contributions of moisture fluxes in different vertical layers to the rainfall amount over the SWTP. Advection of water vapour is reduced by 50% in the whole air column, above 5 km AGL and above 2.5 km AGL, respectively, in these three sensitivity simulations. Radiosonde measurements indicate that the middle and upper troposphere over CEI are nearly saturated (relative humidities of 70–100%) on CS days, whereas non-convective days are rather dry with relative humidities of 20–50% above the boundary layer (Supplementary Fig. 10). For simplicity, we reduce the specific humidity above 5 km AGL by 50% to mimic the situation without convection. This simulation is intended to quantify the contribution of up-and-over transport. The second simulation, in which we reduce moisture advection by 50% above 2.5 km AGL, is intended to quantify the contribution of upslope transport. The final simulation, in which we reduce moisture advection throughout the column by 50%, is intended to quantify the total contribution of transport across the southern boundary.

### Isotope observations

The TNIP includes more than 30 observational stations on the TP, with the longest records dating back to the 1960s and the most recent records collected in 2007 (ref. 27). We select event-based precipitation data from four TNIP stations and one ITP station that measured δ^18^O ratios in precipitation over the southern or SWTP during 1998–2007. We select the station at Lulang, located in the southeastern TP, to compare differences in moisture sources for rainfall over the SWTP and the southeastern TP. A total of 98 summer rainfall events corresponding to our categorized CSs were observed at the five selected stations (Supplementary Table 3). Additional details regarding these measurements have been provided by Yao *et al*^27^.

### Isotope theoretical model

The theoretical amount effect is modelled by treating local convection as a Rayleigh distillation process operating on a single parcel, with condensate immediately removed as precipitation after it forms^36^. Isotopic fractionation is assumed to occur at thermodynamic equilibrium. The liquid–ice transition is neglected for simplicity; its inclusion does not affect the qualitative nature of the results. The model includes five free parameters, the surface pressure (set to be 560 hPa) and the initial temperature (ranges from 260 to 280 K), relative humidity (set to 80%), vapour isotopic composition (δ^18^O_v_=–15‰) and mass of the parcel. In the absence of suitable constraints, the last two parameters are used to fit the model to the observations. With the exception of δ^18^O_v_, which acts to shift the model output left and right to lower or higher values of δ^18^O, variation of these parameters only affects the quantitative end points and does not affect the qualitative output of the model. The shapes of the theoretical curves, which are the basis for our conclusion that moisture source and transport effects govern the differences between intrusive and non-intrusive cases, are unaffected by these variations. The most important source of uncertainty in this model and its interpretation is the role of post-condensation exchange, as equilibration and partial evaporation of raindrops falling through an unsaturated cloud layer can have important impacts on the isotopic composition of precipitation at the ground^37^. These effects are dependent on raindrop size, relative humidity below the cloud base and the depth of the unsaturated layer, and are a key component of the overall amount effect. Reanalysis data indicate that the relative humidity of near-surface air over the SWTP is generally higher during intrusive cases than during non-intrusive cases. Given similar raindrop sizes, post-condensation exchange will therefore tend to increase δ^18^O in precipitation more during non-intrusive cases than during intrusive cases; however, the opposite would need to be true for post-condensation exchange to explain the deviation of the intrusive observations from the theoretical curve. Although the effects of post-condensation exchange likely cause us to overestimate δ^18^O_v_ relative to its actual value, they are unlikely to affect our main conclusion.

### Backward trajectory analysis

Trajectories are calculated using version 9.02 of the FLEXPART particle dispersion model^34^ driven by ERA-Interim reanalysis and forecast fields^35^ at 1° × 1° horizontal resolution and 3-h temporal resolution. About 50,000 particles filling the domain 30–33° N, 80–84° E and 100–8,000 m AGL are initialized at 18UTC 12 September 2002 and tracked backward in time until 12UTC 8 September 2002 (102 h), with particle locations output hourly. To properly account for the complex topography and relative frequency of moist convection in this region, we conduct these simulations with parametrizations of moist convection, sub-grid scale turbulence and mesoscale velocity fluctuations enabled.

## Additional information

**How to cite this article:** Dong, W. *et al.* Summer rainfall over the southwestern Tibetan Plateau controlled by deep convection over the Indian subcontinent. *Nat. Commun.* 7:10925 doi: 10.1038/ncomms10925 (2016).

## Supplementary Material

Supplementary Information Supplementary Figures 1-10, Supplementary Tables 1-4 and Supplementary References.
