# Supplementary material for: Summer rainfall over the southwestern Tibetan Plateau controlled by deep convection over the Indian subcontinent
Source: Nat Commun. 2016 Mar 7;7:10925. doi: 10.1038/ncomms10925 (PMC4786685; doi:10.1038/ncomms10925)
Supplement: Supplementary Information — Supplementary Figures 1-10, Supplementary Tables 1-4 and Supplementary References. [file ncomms10925-s1.pdf]

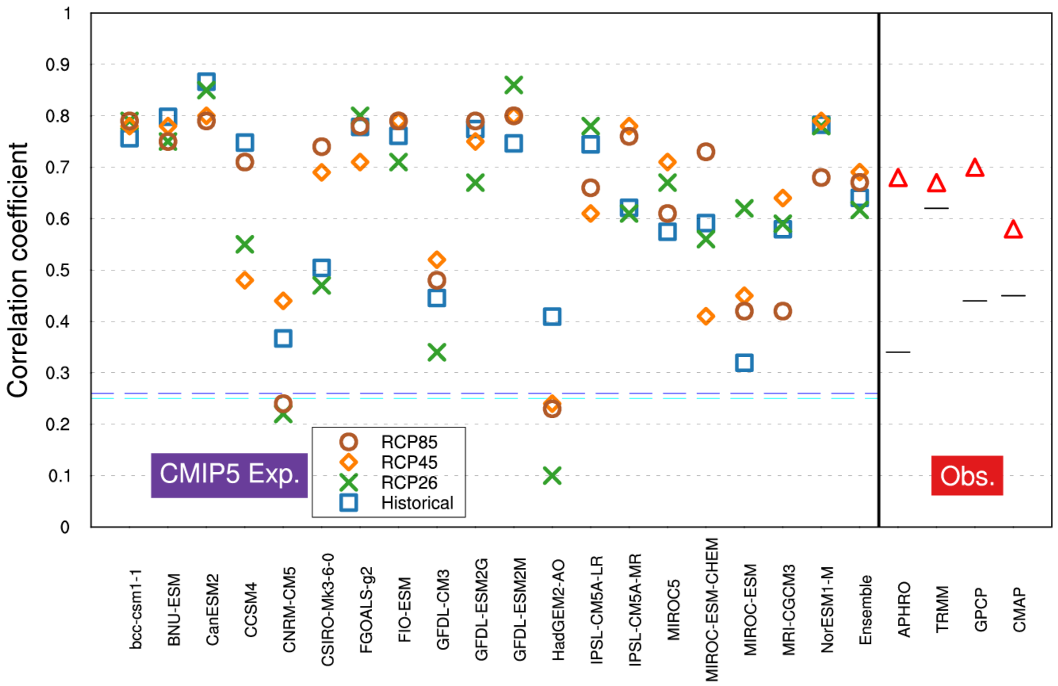

Supplementary Figure 1. Summer rainfall correlations. Correlation coefficients between summer rainfall over the SWTP and summer rainfall over CEI from 19 CMIP5 models (left) and four observational data sets (right). The region definitions and methodology are identical to that used for Fig. 2a (see Methods for details), although the time period varies: 1901–2005 for CMIP5 historical experiments; 2006–2099 for the CMIP5 RCP2.6, RCP4.5 and RCP8.5 experiments; 1951–2007 for APHRODITE; 1998–2013 for TRMM; 1979–2010 for CMAP; and 1979–2012 for GPCP. Dashed lines indicate the 99% confidence level for CMIP5 results, with cyan for historical simulations and violet for future projections. Black dashes indicate the 99% confidence level for each observational data set. Data sources and other information are listed in Supplementary Table 1 for CMIP5 models and Supplementary Table 2 for observational data sets.

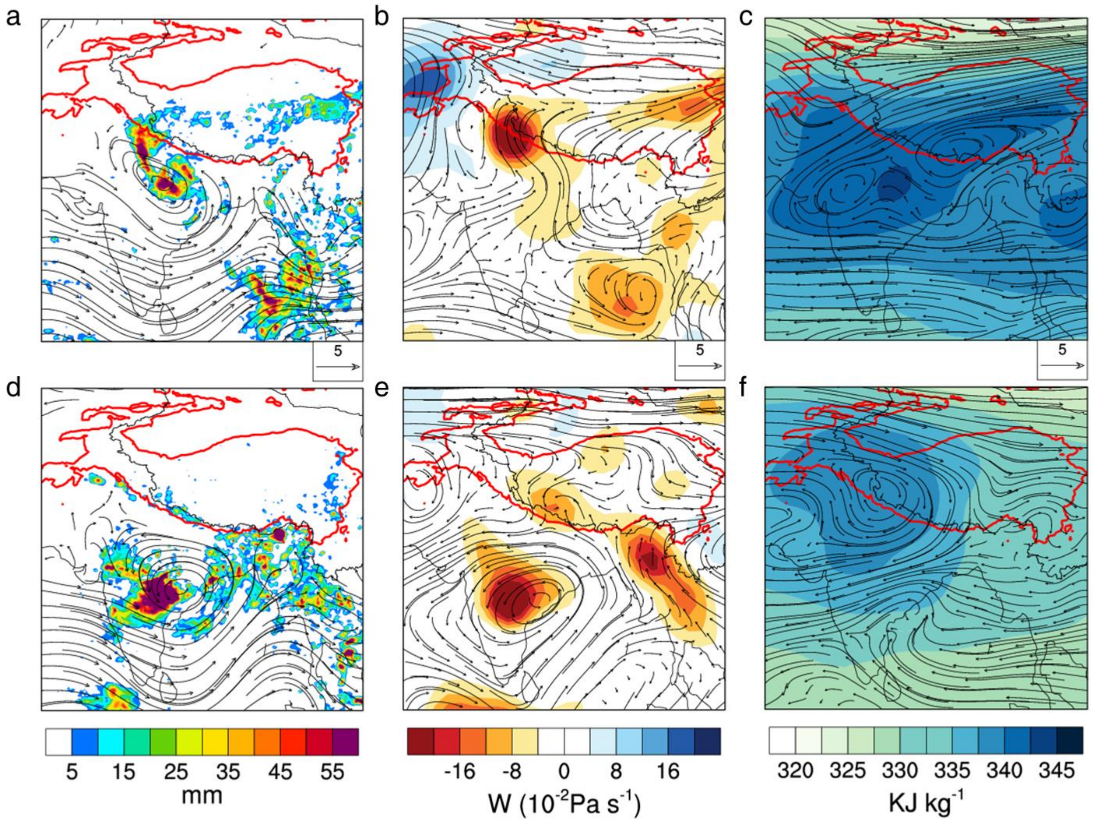

Supplementary Figure 2. Example intrusive and non-intrusive CSs. a, Precipitation overlaid by 850 hPa winds, **b,** vertical velocity (shading) and horizontal winds at 500 hPa and **c,** moist static energy (shading) and horizontal winds at 300 hPa for a typical intrusive CS (12 September 2002). **d-f**, As in a–c, but for a typical non-intrusive CS (23 August 2002). Precipitation data is from TRMM[3]; all other fields are from the ERA-Interim reanalysis[6]. Precipitation amounts less than 5 mm are not shown in **a** and **d**.

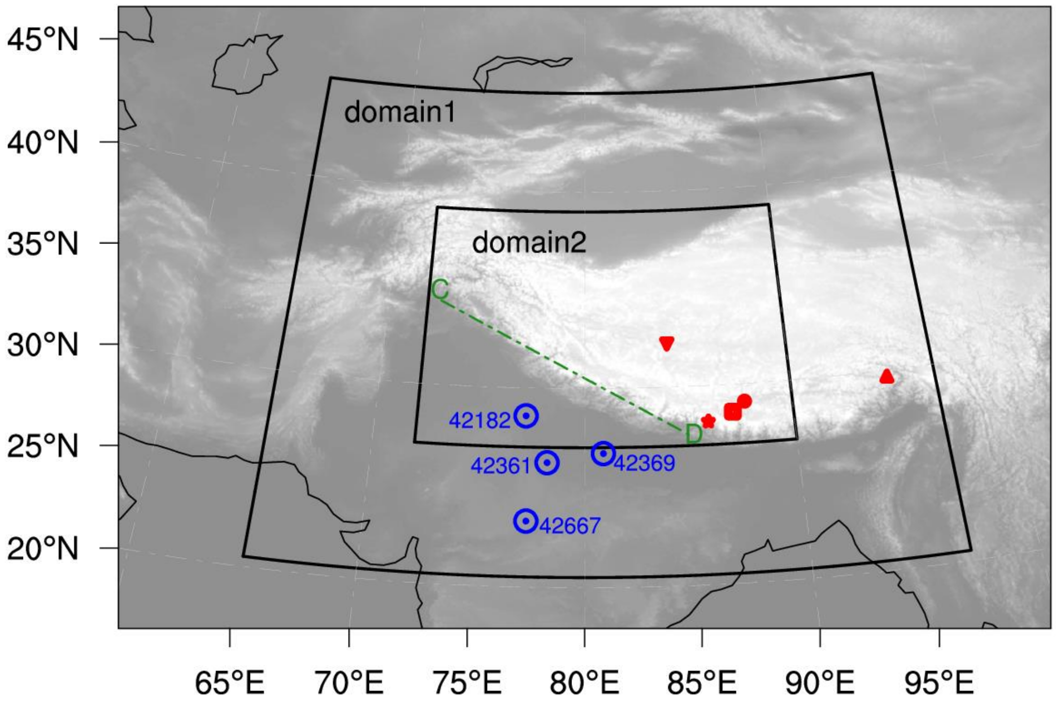

Supplementary Figure 3. Model domains and observation stations. WRF model domains (horizontal resolutions of 27 km in domain 1 and 9 km in domain 2, respectively). The red markers indicate the locations of the five isotopic observation stations (Supplementary Table S3) and the blue markers with station number indicate the location of four radiosonde stations used in Supplementary Figure 10. The green dashed line indicates the cross section in Supplementary Figure 5.

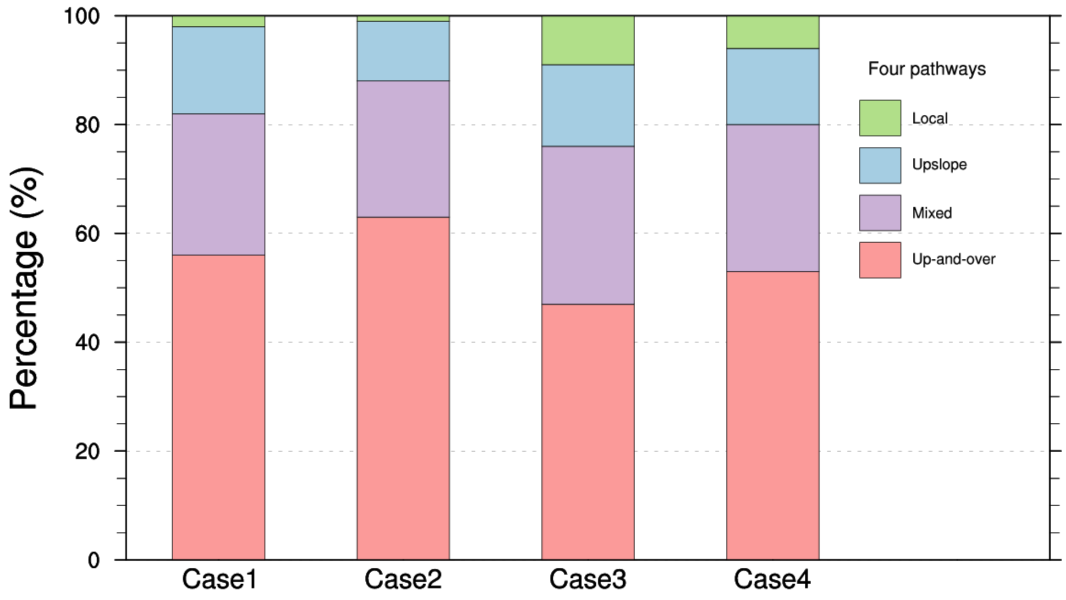

Supplementary Figure 4. Fractions of rainfall over SWTP attributed by the different moisture pathways in WRF simulations. Bars with different color indicate four relevant moisture sources (i.e. up-and-over, mixed, upslope, and local).

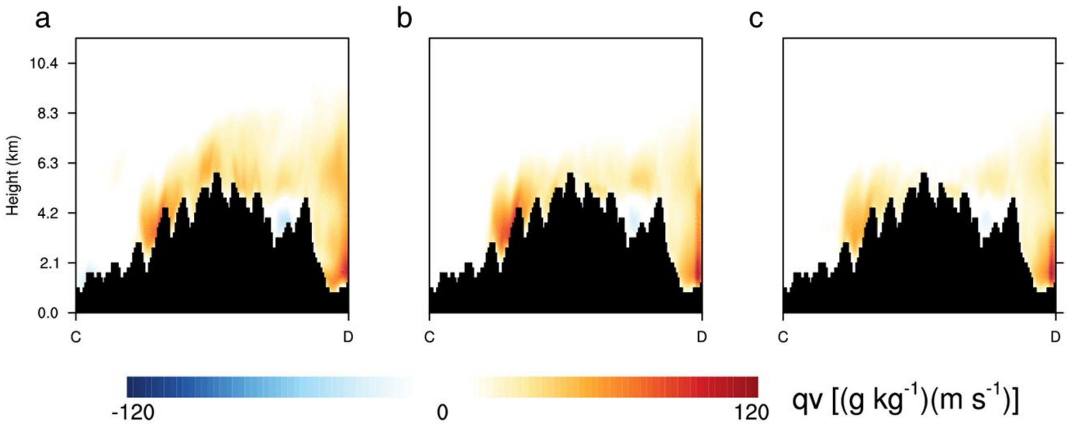

Supplementary Figure 5. Cross-section of meridional moisture transport on 12 Sep. 2002 of, a. CNTL **b.** EXP1**, c.** EXP2, simulations along *C-D* profile in Supplementary Figure 3.

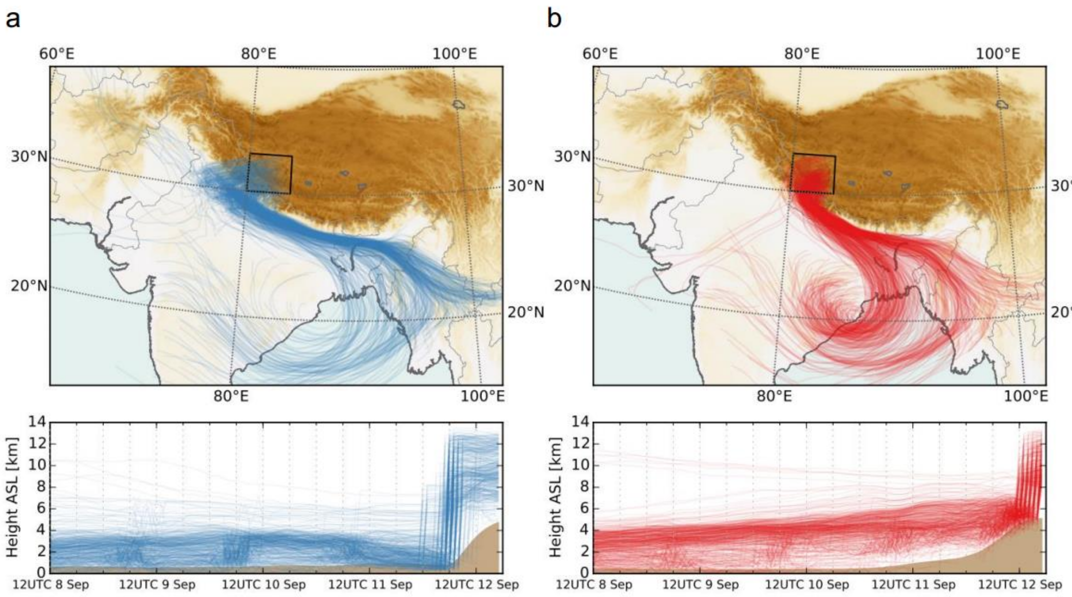

Supplementary Figure 6. Backward trajectories for an intrusive rain event. a, Horizontal and vertical locations of "up-and-over" particles and **b**, horizontal and vertical locations of "local convection" particles. Brown shading in the lower panels shows the mean surface topography beneath the particles. Up-and-over particles are defined as particles that experienced a change in vertical position of 5000 m h^{-1} or more over the Indian subcontinent (surface topography less than 2500 m above sea level) between 00UTC and 18UTC 12 September 2002. Local convection particles are defined as particles with trajectories that experienced a change in vertical position of 3000 m h^{-1} or more over the SWTP (surface topography more than 4000 m above sea level) between 12UTC and 18UTC 12 September 2002. Based on these definitions, the up-and-over and local convection subsets each account for approximately 1% of the total particles. All trajectories were integrated backward for 102 h from 18UTC 12 September 2002 using version 9.02 of the FLEXPART particle dispersion model[11] driven by 3-hourly ERA-Interim reanalysis and forecast fields[6].

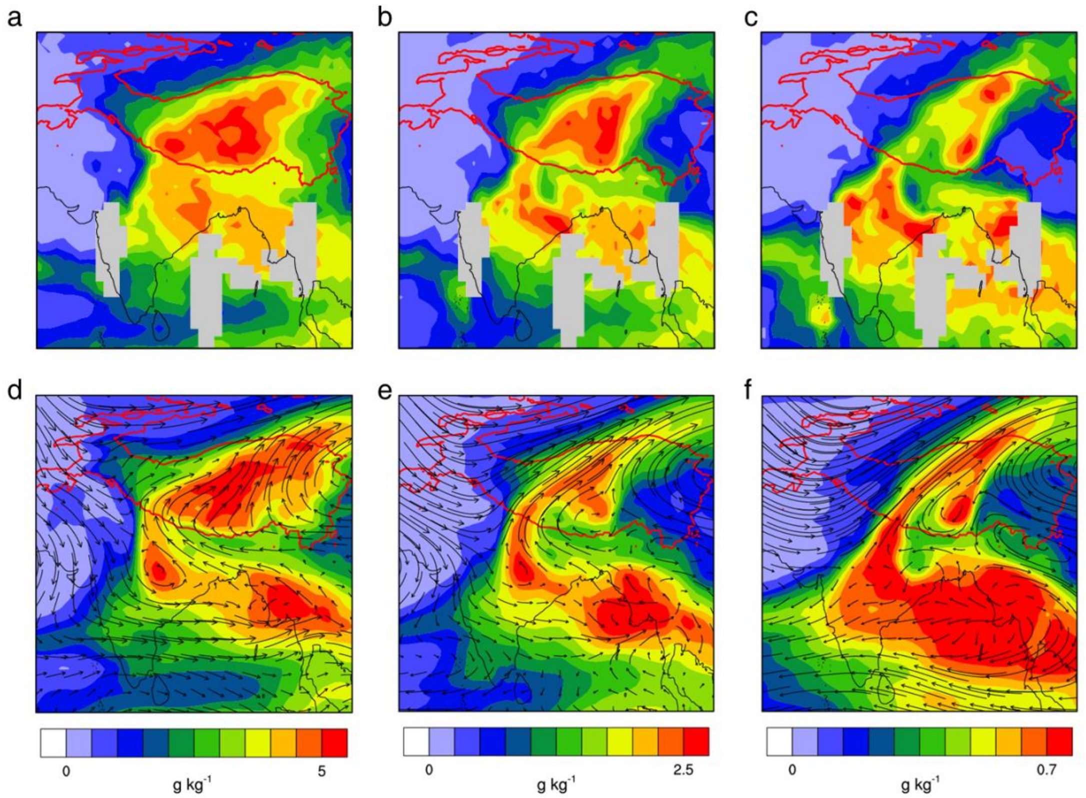

Supplementary Figure 7. Moisture distribution during an episode of intrusive rain event. Horizontal distributions of water vapour during an intrusive CS during 5-8 September 2002 at **a,** 500 hPa, **b,** 400 hPa and **c,** 300 hPa based on twice-daily AIRS observations at 1 °×1 ° resolution. **d–f**, As in **a–c**, but based on four times-daily ERA-Interim reanalysis data[6] and with isobaric winds overlaid. Gray regions in **a–c** indicate missing data.

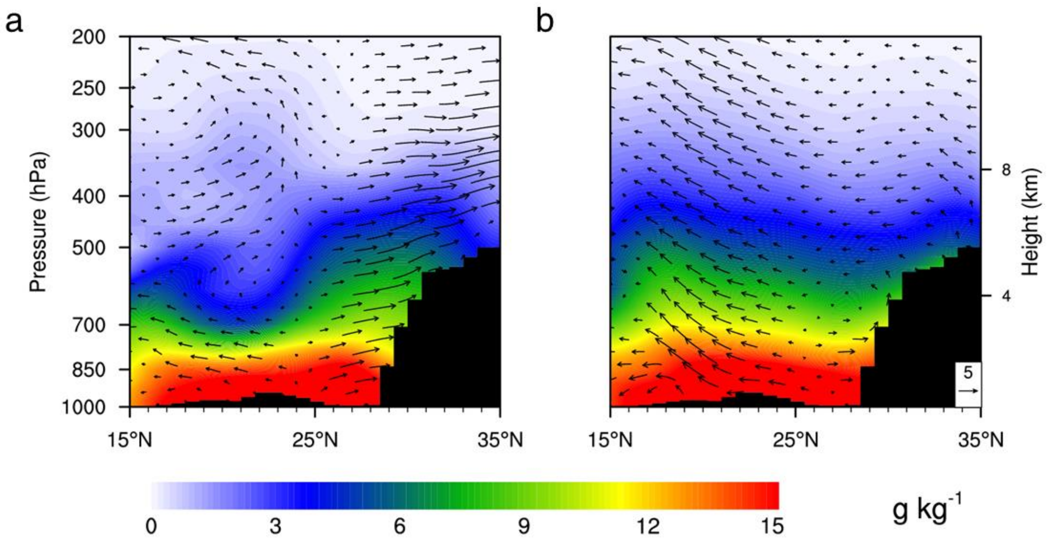

Supplementary Figure 8. Transects of total water content. Cross-sections of meridional and vertical winds with total water content (water vapour plus liquid and ice water content) along 80 E longitude during **a,** a typical intrusive case (12 September 2002) and **b,** a typical non-intrusive case (23 August 2002). All data are from the ERA-Interim reanalysis[6]. Black shading indicates the surface topography along the 80 E transect.

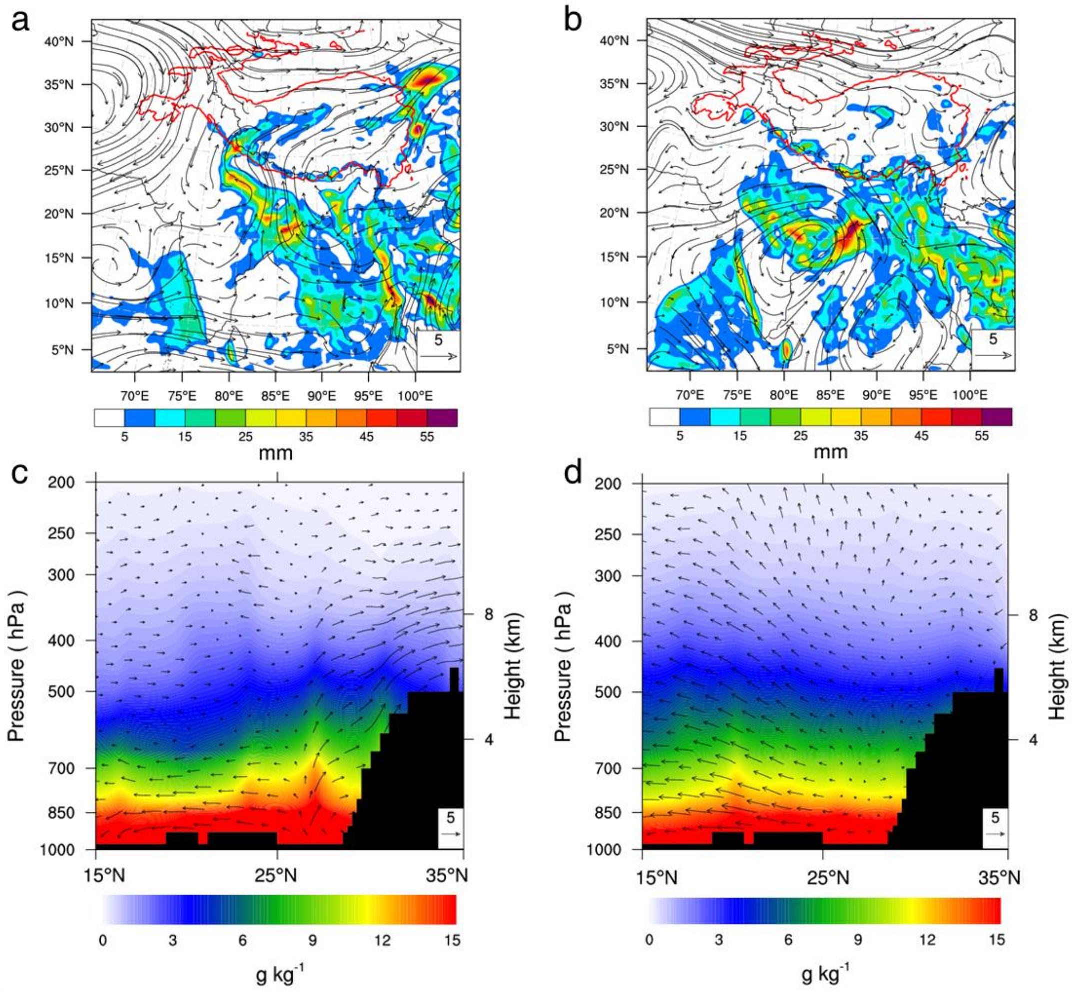

Supplementary Figure 9. Precipitation, circulation and total water profiles from HAR. a, Rainfall distribution and 500 hPa isobaric winds and **c,** vertical profile of total water content and winds along the 80 °E longitude from the High-Asia Refined analysis (HAR) at 30-km resolution for a typical intrusive case (12 September 2002). **b** and **d**, As in **a** and **c**, but for a typical non-intrusive case (23 August 2002). HAR data, which have been prepared using a dynamical downscaling method, have been shown to be reliable over the Tibetan Plateau[12].

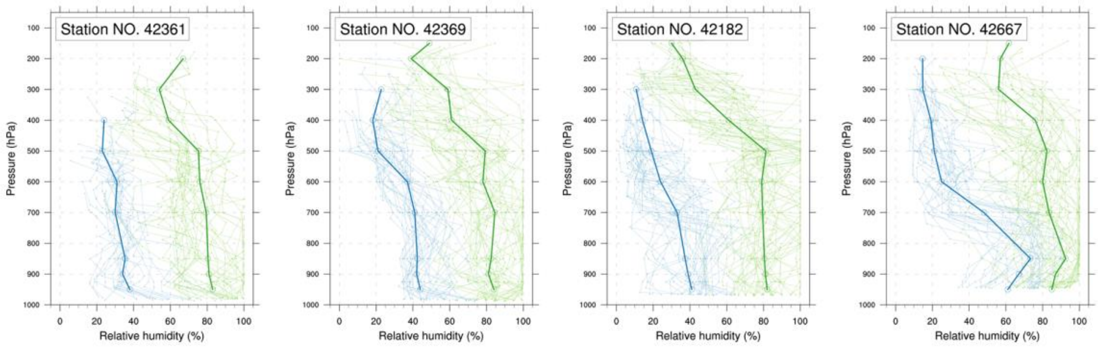

Supplementary Figure 10. Profiles of relative humidity from radiosonde measurements. Light green (blue) lines denote relative humidity profiles on convective (non-convective) days over CEI during the summer in 2002 (See Figure 2b). Dark lines denote the averaged relative humidity after interpolating to standard levels, respectively. The locations of the four stations are marked in Supplementary Figure 3. Radiosonde observations are from the Integrated Global Radiosonde Archive (IGRA), which is available at https://www.ncdc.noaa.gov.

Supplementary Table 1. List of Coupled Model Intercomparison Project phase 5 (CMIP5)[1] models used in this study, with contributor information, full expansion and horizontal grid size (longitude × latitude)

CMIP5 models & Resolution	Sponsor, Country	Model Expansion
bcc-csm1-1 (128×64)	Beijing Climate Center (BCC), China	Beijing Climate center, Climate System Model, version 1.1
BNU-ESM (128×64)	Beijing Normal University (BNU), China	Beijing Normal University-Earth System Model
CanESM2 (128×64)	Canadian Centre for Climate Modeling and Analysis (CCCMA), Canada	Second Generation Canadian Earth System Model
CCSM4 (288×192)	National Center for Atmospheric Research (NCAR), United States	Community Climate System Model, version 4
CNRM-CM5 (256×128)	Centre National de Recherches Meteorologiques (CNRM), France	Centre National de Recherches Meteorologiques Coupled Global Climate Model, version 5
CSIRO-MK3-6-0 (192×96)	Commonwealth Scientific and Institute Research Organisation (CSIRO), Australia	Commonwealth Scientific and Industrial Research Organisation Mark, version 3.6.0
FGOALS-g2 (128×60)	Institute of Atmospheric Physics (IAP), China	Flexible Global Ocean-Atmospheric-Land System Model gridpoint, second spectral version
FIO-ESM (128×64)	State Oceanic Administration (SOA), China	the First Institute of Oceanography-Earth System Model
GFDL-CM3 (144×90)	Geophysical Fluid Dynamics Laboratory (GFDL), United States	Geophysical Fluid Dynamics Laboratory Climate Model, version 3
GFDL-ESM2G (144×90)	—	Geophysical Fluid Dynamics Laboratory Earth System Model with Generalized Ocean Layer Dynamics (GOLD) component (ESM2G)

GFDL-ESM2M (144×90)	—	Geophysical Fluid Dynamics Laboratory Earth System Model with Modular Ocean Model 4 (MOM4) component (ESM2M)
HadGEM2-AO (192×145)	Met Office (UKMO), UK	Hadley Centre Global Environment Model, version2-Atmosphere-Ocean
IPSL-CM5A-LR (96×96)	L'Institut Pierre-Simon Laplace (IPSL), France	L'Institut Pierre-Simon Laplace Coupled Model, version 5, coupled with NEMO, low resolution
IPSL-CM5A-MR (144×143)	—	L'Institut Pierre-Simon Laplace Coupled Model, version 5, coupled with NEMO, mid resolution
MIROC5 (256×128)	Model for Interdisciplinary Research on Climate (MIROC), Japan	Model for Interdisciplinary Research on Climate, version 5
MIROC-ESM-CHEM (128×64)	—	Model for Interdisciplinary Research on Climate, Earth System Model, Chemistry Coupled
MIROC-ESM (128×64)	—	Model for Interdisciplinary Research on Climate, Earth System Model
MRI-CGCM3 (320×160)	Meteorological Research Institute (MRI), Japan	Meteorological Research Institute Coupled Atmosphere-Ocean General Circulation Model, version 3
NorESM1-M (144×96)	Norwegian Climate Center (NCC), Norway	Norwegian Earth System Model, version 1 (intermediate resolution)

*All CMIP5 data are acquired from http://pcmdi9.llnl.gov/.

Supplementary Table 2. Datasets used in this study with full expansion and reference.

Dataset	Resolution & Time Period	Expansion and Reference
APHRODITE (http://www.chikyu.ac.jp/precip/)	0.25 °×0.25 ° 1951–2007	Asian Precipitation - Highly-Resolved Observational Data Integration Towards Evaluation of water resources[2]
TRMM (http://pmm.nasa.gov/data-access/downloads/trmm)	0.25 °×0.25 ° 1998–2013	Tropical Rainfall Measurement Mission[3]
GPCP (http://www.esrl.noaa.gov/psd/data/gridded/data.gpcp.html)	2.5 °×2.5 ° 1979–2012	Global Precipitation Climatology Project[4]
CMAP (http://www.esrl.noaa.gov/psd/data/gridded/data.cmap.html)	2.5 °×2.5 ° 1979–2010	CPC Merged Analysis of Precipitation[5]
ERA-Interim (http://apps.ecmwf.int/datasets/data/interim-full-daily/)	1.5 °×1.5 ° 1979–2013	ECMWF third generation Reanalysis for the global atmospheric condition[6]
CLAUS (http://browse.ceda.ac.uk/browse/badc/claus)	0.3 °×0.3 ° 1983–2009	Cloud Archive User Service[7]
AIRS (http://acdisc.sci.gsfc.nasa.gov/opendap/Aqua_AIRS_Level3/AIRX3STD.006/)	1 °×1 ° 2002–2015	Atmospheric Infrared Sounder[8], version 6[9]

Supplementary Table 3. Detailed list of isotope observations and station locations.

No.	Station	Latitude (°,′N)	Longitude (°,′E)	Altitude (m)	Database
1	Dingri	28,39	87,07	4330	TNIP
2	Larzi	29,05	87,41	4000	TNIP
3	Nyalam	28,11	85,58	3810	TNIP
4	Lulang	29,46	94,44	3327	TNIP
5	Gaize	32,18	84,04	4430	TNIP/ITP[10]
**Station No.**	**$\delta^{18}O$ (‰)**	**Precip (mm)**	**Station No.**	**$\delta^{18}O$ (‰)**	**Precip (mm)**
1*	-9.739	45.1	3*	-13.064	30.3
1*	-19.618	80.9	3*	-16.864	98.1
1*	-22.031	171.0	3*	-13.251	20.9
1*	-20.230	50.8	3*	-13.391	39.8
1*	-16.289	7.5	3*	-13.471	100.6
1*	-15.018	18.6	3*	-12.684	48.3
1*	-18.291	87.5	3*	-10.067	56.1
1*	-20.869	69.0	3*	-10.695	37.8
1*	-19.413	54.8	3	-10.141	14.2
1*	-18.204	117.8	3	-8.326	16.1
1*	-21.648	70.2	3	-9.425	10.3
1*	-11.874	28.5	3	-15.858	5.4
1*	-15.745	41.4	3	-14.211	15.2
1*	-16.070	62.0	4*	-12.004	74.4
1*	-18.542	76.1	4*	-15.251	111.4
1	-6.131	8.3	4*	-16.108	94.6
1	-4.316	6.1	4*	-21.329	37.6
1	-10.462	8.7	4	-9.917	27.2
1	-3.119	1.6	4	-8.832	23.1
1	-12.719	15.6	4	-12.826	25.5
2*	-13.061	15.8	5*	-13.179	53.3
2*	-20.704	93.0	5*	-11.483	24.4
2*	-21.271	79.3	5*	-28.010	30.3
2*	-13.467	27.9	5*	-12.004	74.4
2	-7.153	8.2	5*	-15.251	111.4
2	-7.311	9.1	5*	-16.108	94.6
2	-8.462	10.3	5*	-21.329	37.6
2	-9.435	11.1	5*	-19.373	61.3
2	-10.125	12.3	5*	-14.714	27.4
2	-8.632	13.7	5*	-15.246	34.2
2	-15.624	11.3	5	-4.129	1.2

3*	-17.729	13.3	5	-3.307	1.1
3*	-18.988	31.9	5	-5.462	1.5
3*	-18.477	69.3	5	-5.728	1.7
3*	-18.154	50.7	5	-6.124	2.3
3*	-17.317	47.6	5	-6.421	3.9
3*	-11.156	59.0	5	-7.117	4.9
3*	-17.584	54.7	5	-7.342	4.7
3*	-16.173	41.1	5	-7.791	4.1
3*	-16.367	18.1	5	-9.931	0.4
3*	-18.489	153.6	5	-9.842	2.8
3*	-21.314	132.2	5	-13.935	1.4
3*	-17.079	37.8	5	-11.753	7.3
3*	-16.400	49.2	5	-13.231	6.7
3*	-14.960	96.2	5	-7.935	4.3
3*	-14.333	54.4	5	-12.251	6.3
3*	-15.713	67.3	5	-13.533	17.4
3*	-18.120	77.6	5	-14.553	12.3
3*	-21.863	177.4	5	-12.252	21.3

Note: Station No. with an asterisk indicates the intrusive CS event

Supplementary Table 4. Detailed information of WRF simulations.

**Version**	WRF3.7.1
**Map and grids**	
Map projection	Lambert conformal
Center point of domain	33 °N, 87 °E
Number of vertical layers	40
Horizontal grid spacing	27 km and 9 km
Static geographical fields	U.S. Geological Survey (USGS)
Boundary and initial conditions	6-hour ERA-Interim[6] from the European Centre for Medium Range Forecasts (ECMWF)
**Nesting strategy**	
Nesting	One-way nesting using ndown method
**Simulated cases**	
Case1	12. Sep. 2002
Case2	6. Sep. 2002
Case3	16. Aug. 2003
Case4	17. Aug. 2004
**Physics Options**	
Microphysics	SBU-YLin scheme
Shortwave radiation	Dudhia scheme
Longwave radiation	RRTM scheme
Surface layer	Revised MM5 Monin-obukhov scheme
Land surface	Unified Noah land surface model
Planetary Boundary layer	YSU scheme
Cumulus parameterization	Kain-Fritsch scheme

Supplementary References

1. Taylor, K. E., Stouffer, R. J. & Meehl, G. A. An overview of CMIP5 and the experiment design. *Bull. Amer. Meterol. Soc.* **93**, 485-498 (2012).
2. Yatagai, A. *et al*. APHRODITE: Constructing a long-term daily gridded precipitation dataset for Asia based on a dense network of rain gauges. *Bull. Amer. Meterol. Soc*. **93**, 1401-1415 (2012).
3. Huffman, G. J. *et al.* The TRMM Multisatellite Precipitation Analysis (TMPA): Quasi-global, multiyear, combined-sensor precipitation estimates at fine scales. *J. Hydrometeor.* **8**, 38–55 (2007).
4. Adler, R. F. et al. The version 2 Global Precipitation Climatology Project (GPCP) monthly precipitation analysis (1979-Present). *J. Hydrometeor.* **4**, 1147-1167 (2003).
5. Xie, P. & Arkin, P. A. Global precipitation: a 17-year monthly analysis based on gauge observations, satellite estimates, and numerical model outputs. *Bull. Amer. Meteor. Soc.* **78**, 2539-2558 (1997).
6. Dee, D. P. *et al*. The ERA-Interim reanalysis: configuration and performance of the data assimilation system. *Quart. J. Roy. Meteorol. Soc.***137**, 553–597 (2011).
7. Hodges, K. I., Chappell, D. W. Robinson, G. J. & Yang, G. An improved algorithm for generating global window brightness temperatures from multiple satellite infrared imagery. *J. Atmos. Oceanic Technol.* **17**, 1296–1312 (2000).
8. Aumann, H. H. *et al*. AIRS/AMSU/HSB on the Aqua mission: Design, science objectives, data products, and processing systems. *IEEE T. Geosci. Remote* **41**,

253–264 (2003).

9. Tian, B. *et al.* AIRS/AMSU/HSB Version 6 Level 3 Product User Guide. Tech. Rep., Jet Propulsion Laboratory, Pasadena, California (2013).

10. Tian, L. *et al*. Stable isotopic variations in west China: A consideration of moisture sources. *J. Geophys. Res.* **112**, D10112 (2007).

11. Stohl, A. *et al*. Technical note: The Lagrangian particle dispersion model FLEXPART version 6.2. *Atmos. Chem. Phys.* **5**, 2461–2474 (2005).

12. Maussion, F. *et al.* Precipitation seasonality and variability over the Tibetan Plateau as resolved by the High Asia Reanalysis. *J. Clim.* **27**, 1910-1927 (2014).
